# Biomarkers of Metabolic Disorders: Diagnostic and Prognostic Values, and Insights into the Pathogenesis

**DOI:** 10.1155/2014/586272

**Published:** 2014-06-05

**Authors:** Cheng Hu, Jiarui Wu, Wei Jia

**Affiliations:** ^1^Department of Endocrinology and Metabolism, Affiliated Sixth People's Hospital, Shanghai Jiao Tong University, Shanghai 200233, China; ^2^Institute of Biochemistry and Cell Biology, Shanghai Institutes for Biological Sciences, Chinese Academy of Sciences, Shanghai 200031, China; ^3^Cancer Epidemiology, University of Hawaii Cancer Center, Honolulu, HI 96813, USA; ^4^Center for Translational Medicine, Shanghai Jiao Tong University Affiliated Sixth People's Hospital, Shanghai 200233, China

In recent decades, overwhelming evidence has indicated that metabolic disorders, including obesity, diabetes, dyslipidemia, and nonalcohol fatty liver disease, are the leading cause of disability and death. However, the pathogenesis of metabolic disorder and its chronic complications involves multiple biological pathways and is largely unknown. Biological markers can be evaluated as indicators of pathogenic processes or pharmacological responses to therapeutic interventions. Taking type 2 diabetes, for example, though some clinical phenotypes such as obesity and impaired glucose tolerance are confirmed to be risk factors for the disease, the genetic information can be detected as earlier indicators for prevention and therapy. Due to the heterogeneity of the metabolic diseases in different ethnics, it is imperative to investigate the genetic characteristics in Chinese population. Therefore, the papers selected for this special issue mainly focused on exploring the genetic predisposition and pathogenesis for obesity and type 2 diabetes and their complications. The selected topics and papers are not exhaustive; they do represent the rich and many-faceted knowledge that we have the pleasure of sharing with the readers. We would like to thank the authors and reviewers for their excellent contributions in assisting us.

This special issue contains seven papers, where two papers discuss genetic susceptibility of type 2 diabetes and diabetic nephropathy and one paper identifies a novel mutation for severe obesity. In addition, one paper investigates the regulation of human fat mass and obesity associated gene (FTO) and one paper evaluates the advantages of different detection methods for ketones and establishes whether detection of the concentration of ketones in the breath is an effective and practical technique. Moreover, one paper performed functional studies to examine therapeutic effects of olmesartan on adipose tissue. Finally one review sheds light on the pathogenesis and its clinical applications of type 2 diabetes systematically.

In the paper entitled “*Association of genetic variants of BMP4 with type 2 diabetes mellitus and clinical traits in a Chinese Han population*,” S. Tang et al. test the impacts of* BMP4* variants on type 2 diabetes in the Chinese including 3,410 diabetic patients and 3,412 normal glucose regulation individuals and finally present a minor effect of* BMP4* variants on glucose metabolism in Chinese population.

In the paper entitled “*Lack of association between TLR4 genetic polymorphisms and diabetic nephropathy in a Chinese population*,” D. Peng et al. investigate the effects of* TLR4* genetic variants on diabetic nephropathy in 1,455 Chinese type 2 diabetic patients and detect no association with the disease.

In the paper entitled “*Genetics of type 2 diabetes: insights into the pathogenesis and its clinical application*,” X. Sun et al. review the major genetic studies on the risk of T2D based on ethnicity and briefly discuss the potential mechanisms and clinical utility of the genetic information underlying T2D.

In the paper entitled “*A novel mutation in leptin gene is associated with severe obesity in Chinese individuals,*” Y. Zhao et al. detect a novel mutation H118L in* leptin* gene in Chinese subjects. This novel mutation might be the casual variant leading to severe obesity; however, functional studies still needed to be confirmed.

In the paper entitled “*CCAAT/enhancer-binding protein *α* is a crucial regulator of human fat mass and obesity associated gene transcription and expression*,” W. Ren et al. aim to study the possible mechanism of how the C/EBP*α* binding site regulates FTOgene expression. They suggest that C/EBP*α* may act as a positive regulator binding to FTO promoter and, consequently, activates the gene transcription.

In the paper entitled “*Breath ketone testing: a new biomarker for diagnosis and therapeutic monitoring of diabetic ketosis*,” Y. Qiao et al. established breath ketone testing as a noninvasive, convenient method for the diagnosis and therapeutic monitoring of diabetic ketosis.

In the paper entitled “*Effects of the angiotensin receptor blocker olmesartan on adipocyte hypertrophy and function in mice with metabolic disorders*,” A. Maeda et al. investigate the therapeutic effects of an AT1R-specific blocker, olmesartan, on genetically obese diabetic KKAy mice and analyze possible effects on adipose issue. They indicate that the blood pressure lowering effect of olmesartan in KKAy mice is associated with improvement in adipocyte dysfunction including suppression of adipocyte hypertrophy and inhibition of adipose IL-6-oxidative stress axis.

These studies will help readers to understand the current status and gain new insights into the genetic traits of metabolic diseases.

*Cheng Hu* *Jiarui Wu* *Wei Jia*

